# Hepatic Surgery in the Ussr

**DOI:** 10.1155/1991/72586

**Published:** 1991

**Authors:** Eduard I. Galperin, Suren R. Karagiulian

**Affiliations:** Department of Hepatobiliary Surgery I.M. Sechenov Moscow Medical Academy Moscow Russia


*HPB Surgery*, 1991, Vol. 4, pp. 191–202
Reprints available directly from the publisher
Photocopying permitted by license only
© 1991 Harwood Academic Publishers GmbH
Printed in the United Kingdom

### HEPATIC SURGERY IN THE USSR

EDUARD I. GALPERIN and SUREN R. KARAGIULIAN,
*Department of Hepatobiliary Surgery, I.M. Sechenov Moscow Medical Academy, Moscow, USSR*

*(Received 11 September 1990)*

The history of hepatic surgery in the USSR goes back to the work of Russian surgeons in the late nineteenth century. The first successful liver resection was performed in Russia by N.V. Sklifosovsky in 1889, further contributions to surgery of the liver were made by M.M. Kuznetsov and V.R. Pensky who suggested the hepatic suture (Figure 1) in 1894 [1,2].

At the same time as the topographical studies of Couinaud [3,4] and Reifferscheid [5] revealed the segmental structure of the liver, a large contribution to the understanding of the surgical anatomy of the liver was made by the anatomical explorations of A. V. Melnikov, [6] who studied the blood supply of the anatomical zones of the liver. G. E. Ostroverkhov and co-workers described in detail the topography and projection anatomy of Glisson's pedicles of the liver's lobes, sectors and segments, and produced a useful and practical monograph on the principles of anatomical lobar liver resections (LR), [7–11]. V. S. Shapkin, produced a classification of anatomical and atypical LR, and together with M. Sh. Izraelashvili, studied in detail the natural history of intrahepatic metastases. Shapkin was one of the first in this country to use intravascular bulb obturation to exclude either the whole liver or a part of it from the circulation while performing LR; [2,12–16] soon others adopted this technique [17–20].

In the USSR hepatic surgery is now one of the most rapidly developing areas of abdominal surgery, making use of modern diagnostic and therapeutic methods. There are specialized units for hepatic surgery at the clinics of research institutes in the large cities of Russia and other union republics. In Moscow: at the National Scientific Centre of Surgical Research (under the general guidance of Academician B. V. Petrovsky); at the I.M. Sechenov Medical Academy in Moscow (Professor E. I. Galperin), at the A. V. Vishnevsky Institute of Surgery (under the general guidance of Academician V. D. Fyodorov); in Leningrad: at the First Medical Institute (Professor A. M. Granov); in Kiev, the Ukraine: at the Scientific Research Institute of Clinical and Experimental Surgery (Academician A. A. Shalimov); in the cities of Kirov, Tomsk, Krasnoyarsk, Novosibirsk in Siberia: teams under the guidance of Professors V. A. Zhuravlev, B. I. Alperovich, R. A. Nikhinson and Yu. M. Lubensky, G. I. Veronsky. Surgical procedures for parasitic diseases and tumours of the liver are widely performed in the large cities of Central Asia (Alma Ata, Kazakhstan; Tashkent, Uzbekistan; Frunze, Kirghizia) and the Caucasus (Tbilisi, Georgia; Baku, Azerbaijan).

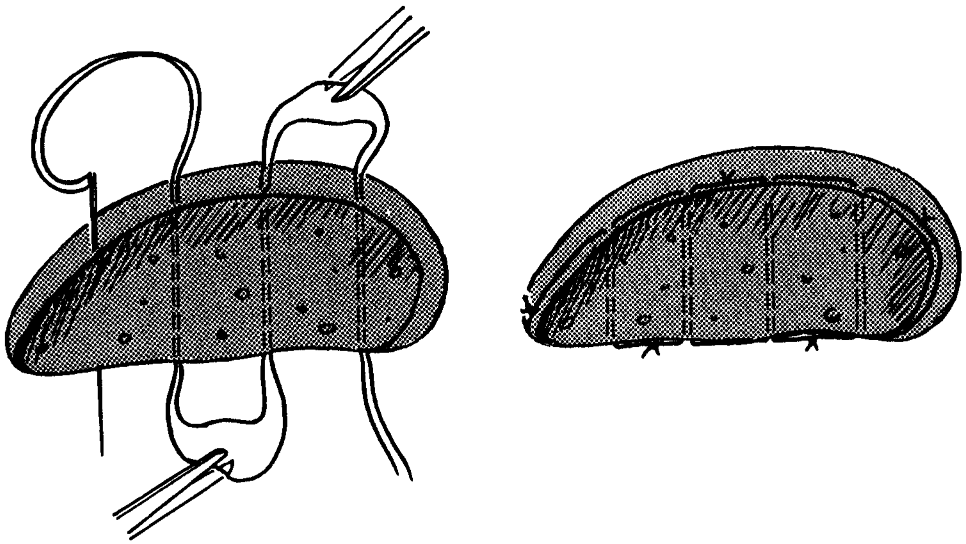

**Figure 1** Hepatic suture suggested by M.M. Kuznetsov and V.R.Pensky[1,2].

Across the vast territory of the Soviet Union there is a different prevalence of the various liver tumours in the different republics and regions. Thus, in the European part of the country the prevalence of primary liver cancer (PLC) is comparatively low, just as throughout Europe, and accounts for 0.4–1.76% of tumours and is found in 0.05–0.57% of all autopsies. In areas of Siberia affected with opisthorchosis, which frequently leads to the development of cirrhosis of the liver, PLC morbidity is 4.47–22.3% of deaths from all malignant tumours. [1,21–24] Hepatocellular adenoma occurs in the USSR much less frequently than in countries where the use of steroid contraceptives is widespread. In the southern and eastern regions of the USSR (republics of Central Asia, the Caucasus, in Eastern and Western Siberia and in the Far East) surgeons very frequently have to deal with hydatid or alveolar echinococcosis of the liver. Statistics show that between several hundred [25–29] and several thousands [30,31] operations for the surgical treatment of these diseases are performed in these regions each year, whereas data coming from the clinics of Moscow, Leningrad and other large cities in the European part of the USSR seldom report more than 30–50 cases, and only specialized hepatological units which receive patients from all over the country have experience in the surgical treatment of 120 to 150 patients per year [32–36].

The major areas of interest in the development of Hepatic Surgery in this country coincide with those under investigation elsewhere: devising techniques for reducing the trauma of liver resection, improving the techniques for performing partial or complete temporary exclusion of the liver from the circulation, investigating better methods of temporary and final hemostasis, and striving to prevent the complications that frequently attend major liver surgery. Non-operative techniques are also under development for the transcutaneous puncture treatment of nonparasitic cysts, abscesses and residual cavities of the liver; and endovascular occlusion of tumoral vessels. All the various facets of liver transplantation are being actively pursued.

The diagnostic techniques used in large hepatology units differ little from those in general use around the world. Ultrasound, computed tomography, magnetic resonance imaging, angiography and fine-needle aspiration biopsy are all in use, the later technique has been especially useful recently in allowing more precise and earlier detection of liver tumours at a preclinical stage [37–45]. Work on the diagnosis of liver tumours has involved the evaluation of: the information provided, the accuracy and the specificity of different investigations and has also assessed the efficiency of rational combinations of diagnostic methods - diagnostic algorithms [37,44,46]. In areas where hydatid disease is endemic ultra sound and CT are commonly used in combination with serological diagnosis techniques such as the indirect hemagglutination or indirect microhemagglutination of an erythrocyte preparation using as antigen fluid from echinococcal cysts of sheep or a specific fraction obtained from the fluid of disc electrophoresis [1,47,48]. The specificity of this reaction with respect to hydatid disease of the liver was 95.3% in an investigation involving 386 samples of heterologous sera. The technique proved effective in a mass screening programme of the population, aimed at detecting early stages of hydatid disease. The specificity of diagnosing hydatid and alveolar disease is also high when using an immunoenzymatic test with hydatid and alveolar preparations - 90.6% and 98.9% respectively when examining sera of 260 patients with hydatid and 210 with alveolar disease. Use of this technique to examine 3,500 rural dwellers in three endemic areas of Yakutia (Eastern Siberia) revealed a significantly high number of people in whom the test was positive 4.2–15.6% compared with a control population of 1,170 Muscovites where only 0.5% were positive [48].

Surgical techniques for the treatment of liver tumours are being developed along several lines. The surgical apprach of choice at the moment is liver resection. A number of surgeons have personal series of more than 100 liver resections performed for various liver tumours (Table 1).

The teams lead by these and other surgeons have suggested a large number of original surgical approaches. The 1970s and 1980s saw a vigorous debate in the medical literature of this country over the relative benefits of anatomical or atypical liver resection, as a result a consensus was reached that at the present time operations on the liver should take into account the blood supply and bile secretion secretion of both the portion that was being removed and of that remaining, but it is also recognised that elements of both surgical approaches may and in some cases,

**Table 1** Liver resection results according to data from leading hepatic surgeons of the USSR

| *Source* | *Total number of LR (lethality, %)* |
|---|---|
| Alperovich B.I. /49/ | 257*(10.5) |
| Veronsky G.I. /19/ | 170*(9.4) |
| Zhuravlev V.A. /50/ | 150*(13.3) |
| Shapkin V.S. /14/ | 180*(15.5) |
| Rustamov I.S. /51/ | 105(6.7) |
| Nikhinson R.A., Lubensky Yu. M., Krakovsky A.I. /28/ | 103*(12.6) |
| Galperin E.I. /52/ | 107(10.3) |

* Surgery performed chiefly for alveolar disease by surgeons in Siberian cities must be used at different stages of the management of a patient [1,49,51]. V. S. Shapkin has been a consistent champion of anatomical liver resection but in his series 45% of operations were atypical, in our series an atypical resection was used in 47.7% of patients chiefly for liver tumours where less than 1 to 3 segments of the liver had to be removed. With a few exceptions [1,49] an increasing number of surgeons agree that anatomic liver resection is the method of choice for the resection of extensive liver tumours especially when preliminary exclusion from the circulation of the lobe sector or segment to be resected makes it possible to reduce the overall blood loss and trauma of the operation.

In the opinion of some authors the anatomical resection is the most appropriate procedure for primary liver cancer (PLC); having studied the routes taken by metasteses from PLC in corrosion casts of the liver Professors V. S. Shapkin and M. Sh. Izraelashvili have convincingly demonstrated that the veins of the gall bladder play a substantial role in the spread of cancer cells from one anatomical half of the liver to the other. It was established that the vessels of the PLC communicate with branches of the portal vein within the liver as well as with the intramural veins of the gallbladder which in turn join the veins of segments, 4, 5 and 8, thereby effecting a link between the intrahepatic networks of the right and left branch of the portal vein [13,16,54]. This is why the authors strictly adhering to the principles of anatomic liver resection in cases of PLC, recommend that hemihepatectomy be started by cholecystectomy so that manipulations of the affected lobe of the liver should not produce spread of cancer emboli to the opposite intact lobe. Using this approach, these authors note that the life of patients for hepatectomy with cholecystectomy is 38.3 plus/minus 4.6 months compared with 21.7 plus/minus 2.1 months in patients having a similar resection without cholecystectomy (statistically significant difference) [54].

The same authors have suggested a technique using an inflatable balloon catheter to obturate the vessels of the lobes and segments of the liver prior to excision thus reducing the trauma and total blood loss during anatomical lever resection [56,56] (Figure 2). On inflating the balloon in the lumen of a lobal or segmental branch of the portal vein not only is the portal flow arrested but, by compressing from within the remaining elements of the portal triad (branches of the hepatic artery and bile duct) against the dense common epifascial sheath enveloping the triad it also produces cessation of arterial blood flow and bile drainage. This ensures a dry surface to the cut surface of the liver when performing liver resection along the ischemic line.

With the same purpose we have described in detail a method of selectively applied ischemia of the liver by digital transhepatic exposure of the hepatic pedicle of the portion of liver to be removed (Figure 3).

The technique comprises four steps, 1) the superficial T-shaped incision of Glisson's capsule overlying the location of the hepatic pedicle, 2) introduction of the surgeons forefinger into the liver parenchyma, oozing from the parenchyma is controlled by clamping the hepatoduodenal ligament, the finger tip can locate the tubular structure of the pedicle which is distinguished by its smooth elastic wall and separated from the friable parenchyma. The finger is then hooked over the pedicle, 3) the pedicle is drawn down through the hepatotomy and temporarily clamped, when the hepatoduodenal ligament is then released to restore blood flow to the rest of the liver, 4) a check is made to ensure that the discoloured ischemic portion corresponds with the region to be excised, the clamp may be moved proximally or

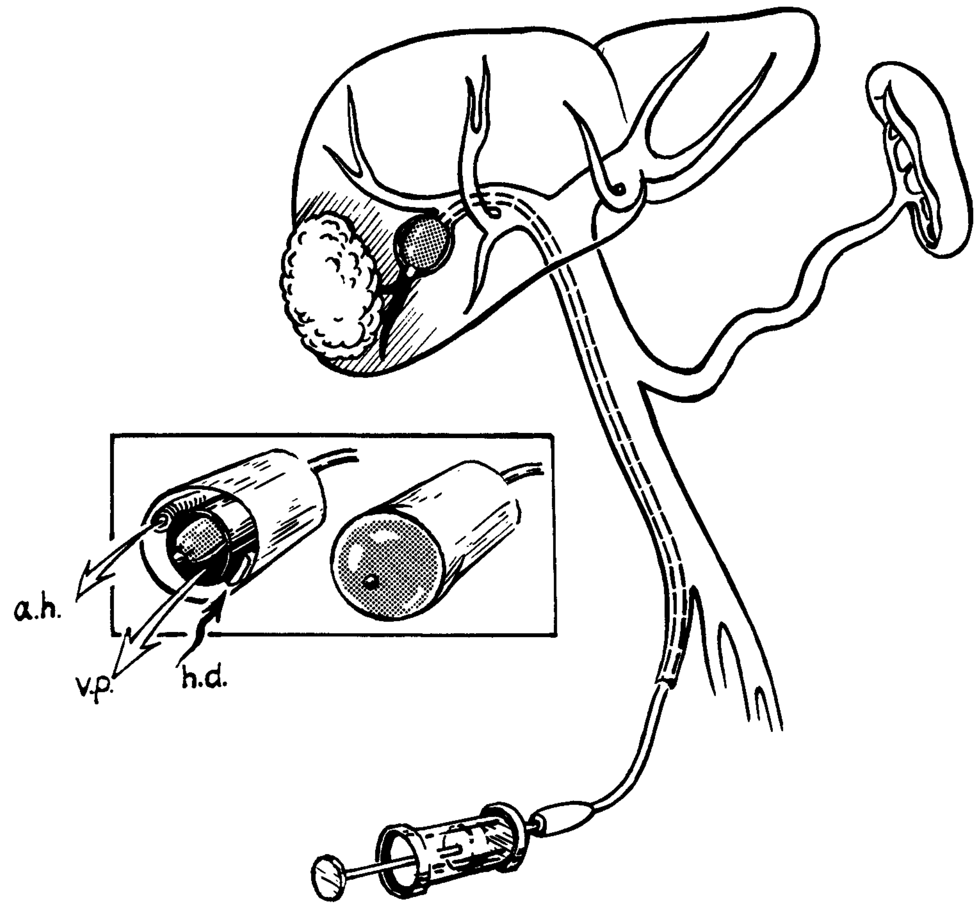

**Figure 2** Scheme of selective obturation of portal triad by using inflatable balloon-catheter[55,56] (see text).

distally along the exposed pedicle to release or clamp the lateral branches as necessary before the resection starts.

There are instruments and devices made in the USSR intended to provide a bloodless and minimally traumatic separation of liver parenchyma: an electrosurgical technique for the dissection of parenchymatous organs had been devised in Kharkov, the Ukraine. This instrument is based on an electrosurgical device with a rotating attachment which, owing to the high frequency current used, bloodlessly dissects the parenchyma and exposes the liver's tubular structures without injuring them [58]. A Soviet model of an ultrasonic surgical aspirator [59] seems to be equally effective. Professor B. I. Alperovich and co-workers in the city of Tomsk, Siberia, have developed an original cryo-ultrasonic scalpel, [1,60] which dissects the liver parenchyma with simultaneous cryocoagulation. Adhesion of the scalpel to the liver is prevented by ultrasonic vibration. Several other instruments for cryosurgery have been developed by the same team [1,60]. Detailed investigations are being

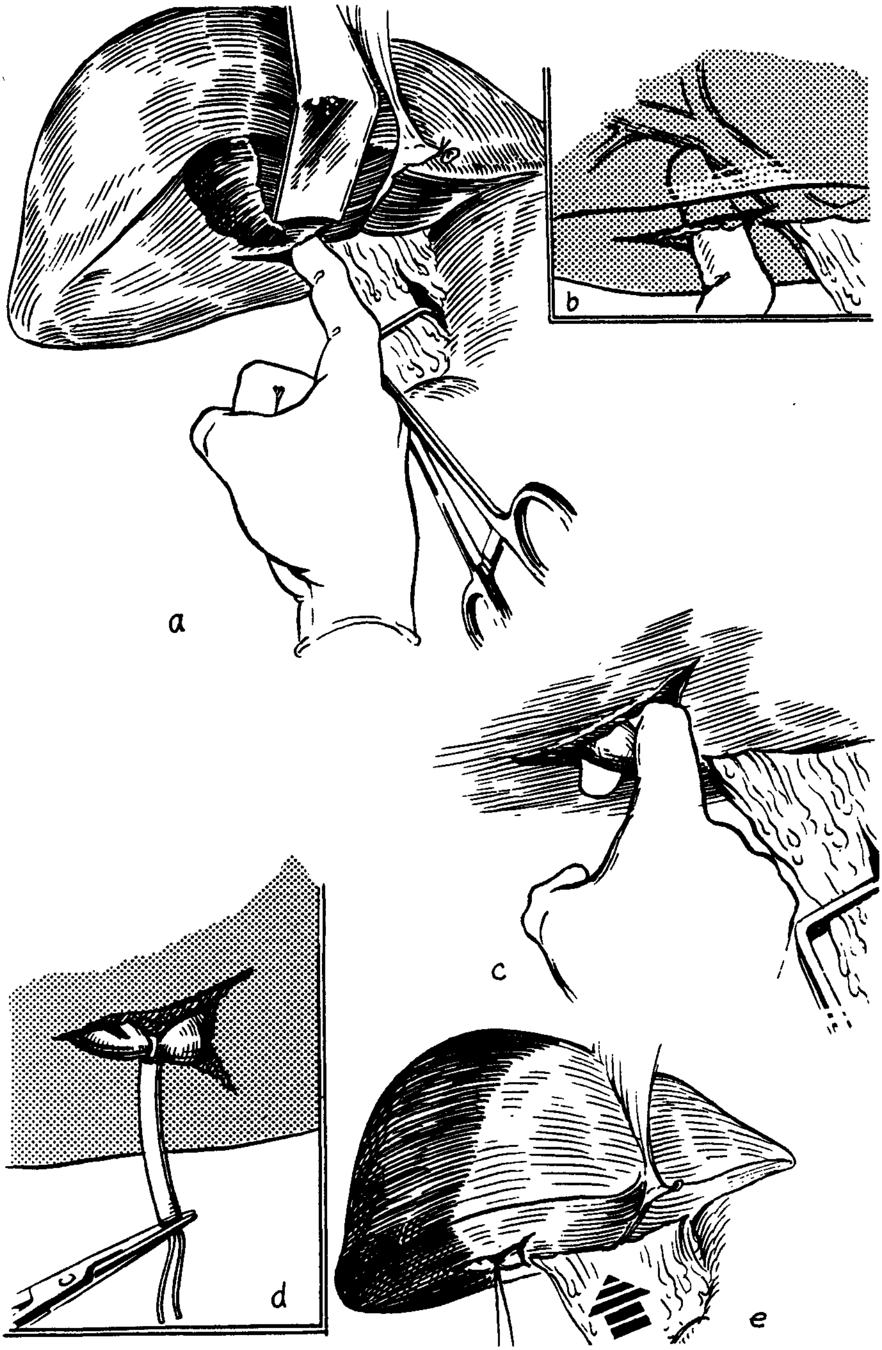

**Figure 3** Stages of transhepatic finger exposure of a pedicle; a to e – steps of procedure[57].

conducted on the use of a low temperature plasma torch for the purpose of hemostasis, [20,61,66] a laser beam, [62,67] and biological glue [68] are also being studied.

Although these instruments are effective in their different ways in separating liver parenchyma there is no radical change in the technique of liver resection [14] details of which have been dealt with in several monographs and scientific papers in recent years, where the substantial personal experience of the authors is combined with research into new approaches to understand and exploit the anatomical arrangements of the liver [1,11,14,18,19,25,60]. Ultra sound or CT controlled transcutaneous punctures of the liver are becoming increasingly important in the armamentarium of techniques for the surgical treatment of liquefied liver tumours, cysts, abscesses and residual cavities. These techniques are growing together into an independent branch of hepatic surgery with its own technical and tactical solutions. Transcutaneous puncture and draining procedures constitute, in our data at least 30% of all the interventions for liver tumours performed at major hepatological centres. In general the techniques used in the USSR do not differ from those used in the rest of the world, stylet-catheters, wire guides and dilators of various calibre, self-fixing drainage tubes of the basket or "pig-tail" type are all used. For sclerosing the cavities of nonparasitic cysts the repeated introduction of a mixture of alcohol and iodine is used as well as the original sclerosing compound [69–71]. Good or satisfactory results are achieved in 80–85% of cases of nonparasitic cysts and abscesses. Increasingly we see reports of the successful use of x-ray guided endovascular occlusion of branches of the hepatic artery used for the treatment of malignant tumours and cavernous hemangiomas [72–74]. This technique is also used for arterial vessels or varices of the esophagus and stomach as a separate and preparatory stage in the surgical treatment of cirrhosis of the liver [75,78] the emboli used are polymer materials in the form of microspheres 0.5–0.75 mm in diameter or cylinders of the same material 8–10 mm long, metal spirals and some other materials are also used [72,73,79].

Transplantation of the liver is a problem which is being resolved with great difficulty in the USSR. The interest here reached its peak in 1970s as a result of the reports of ever more successful liver transplantation (LT) performed by Starzl in the USA and Calne in the UK. Many experimental studies (chiefly in dogs and pigs) were carried out in those years in the study of the technical and functional peculiarities of both orthotopic and heterotopic LT. Special interest in this country was shown for the latter technique, experimental studies were conducted along three main lines, 1) heterotopic LT from an adult donor, 2) heterotopic partial LT from an adult donor and 3) heterotopic transplantation of the liver of newborn puppies into an adult recipient.

The topographical and experimental investigations have resulted in the development of a method of heterotopic extra-peritoneal transplantation of the left lobe of the human liver into the iliac fossa. This is aimed at the functional support of the affected liver of the recipient during its period of regeneration. This method was used in 1977 for the transplantation of the left lobe of a cadaver liver into two patients[83–83]: a 38 year old woman with an inoperable central alveolar echinococcosis had symptoms of protracted obstructive jaundice and chronic hepatic insufficiency, the other patient was a 31 year old male with postnectrotic cirrhosis of the liver and Child's C hepatic insufficiency. An improvement of the clinical and biochemical status of both patients was noted in the immediate post operative period, but, in the first case acute rejection of the transplant occurred on the 10th day, and it was removed on the 17th day, 48 hours after this the patient died; in the second case a thrombosis of the portoiliac venous anastomosis developed which required surgical thrombectomy, the patient died on the 4th day of gastroesophageal haemorrhage from the varices of the esophagus made worse by the increasing hepatic insufficiency. The absence, at that time and up to 1987, of legislation permitting the removal of organs from a heart beating donor, restricted further work in this area to anatomical and experimental investigations and liver transplantation was no longer carried out. Interesting results were obtained from work studying the effects of a supplementary transplant from a newborn puppy to stimulate the regeneration of the recipients liver during a period of ischaemic necrosis. It was found that after the removal of the newborn - transplant, in distinction from the situation with an adult transplant the function of the recipient liver which had undergone profound ischaemic damage as a result of the deflection of the portal blood and ligation of the hepatic artery for 60 minutes, was completely restored after two days and the recipients survived. Yet a transplant from an adult donor merely replaced the functions of the affected liver for a prolonged period, but without providing any stimulating effect on regeneration. This was confirmed by the development of hepatic coma in dogs following the removal of the transplant [84–86].

The passing of a law permitting certain specialized institutions to harvest organs from heart beating donors revived the interest in the problems of liver transplantation and during the first six months of 1990 two teams of Moscow surgeons at the National Surgical Research Centre and the Scientific Research Institute of Transplantology and Artificial Organs, performed six orthotopic liver transplants: four for a malignant tumour and two for cirrhosis.

The 1980s marked a breakthrough for hepatic surgery around the world and this field is becoming increasingly less elitist. Modern technical facilities considerably expand the possibilities for the surgical treatment of liver tumours whilst at the same time diminishing the trauma of the operation. Still a number of problems remain and Soviet surgeons along with their colleagues in other countries are making vigorous contributions to their solution.

### *References*

1. Alperovich B.I. (1983) *Surgery of the Liver*, Tomsk, 350 pp, (in Russian)
2. Shapkin V.S. (1967) *Liver Resection*, Moscow: Meditsina Publishers, 297 pp. (in Russian)
3. Couinaud C. (1957) *Le foie. Etudes anatomiques et chirurgicales*. Paris: Masson
4. Couinaud C. (1981) *Controlled hepatectomies and exposure of the intrahepatic bile ducts. Anatomical and Technical study*. Paris.
5. Reifferscheid M. (1957) *Chirurgie der Leber*. Stuttgart
6. Melnikov A.V. (1956) On Liver Resection. *Khirurgiya*, No. 1, pp.38–47 (in Russian)
7. Ostroverkhov G.E. and Zabrodskaya V.F. (1965) Projection Anatomy of Intrahepatic Bile Ducts. In *Aktualnye voprosy klinicheskoi i eksperementalnoi khirurgii* (topical questions of clinical and experimental surgery). Moscow, pp.309–325 (in Russian)
8. Ostroverkhov G.E., Zabrodskaya V.F. and Umbrumyants O.A. (1966) Topographic details of Glisson's pedicles of the liver's lobes, sectors and segments. - *Transactions of a National Convention of Anatomists, Histologists and Embryologists*. Tbilisi, (in Russian)
9. Ostroverkhov G.E., Zabrodskaya V.G., Zatolokin V.D. (1968) Anatomical substantiation of lobar resections of the liver. Communication 1. *Khirurgiya*, No. 5, p. 151 (in Russian)
10. Ostroverkhov G.E. and Zabrodskaya V.F. (1972) Surgical anatomy of the liver and bile ducts. In *Khirurgicheskaya Anatomiya Zhivota* (Surgical anatomy of the abdomen). Editor Maksimenkov A.N., Leningrad, Meditsina Publishers, pp. 297–384 (in Russian)

11. Ostroverkhov G.E. and Zatolokin V.D. (1984) *Principles of anatomical lobar liver resections*, Moscow, Meditsina Publishers, 114 pp (in Russian)
12. Shapkin V.S. and Malyshev A.F. (1979) Methods of artificial blood circulation in hepatic surgery. *Vestn. khir.*, Vol. 123, No. 10, pp. 23–27 (in Russian)
13. Shapkin V.S. and Izraelashvili M.Sh. (1984) Fundamentals of surgical resection for primary liver cancer. *Methodological recommendations*, Tbilisi, 12 pp. (in Russian)
14. Shapkin V.S. (1986) Liver resection: selection of method and technique. *Khirurgiya*, No. 2, pp. 3–7 (in Russian)
15. Izraelashvili M.Sh. (1975) Exsanguination of one anatomical half of the liver by ligating the appropriate Glisson's pedicle. In *Aktualnye voprosy patologii pecheni i zhelchnykh protokov* (Topical problems of hepatobiliary pathology). Tbilisi, pp.12–13 (in Russian)
16. Izraelashvili M.Sh. (1986) Clinicomorphological aspects of liver resection in benign and malignant tumours. *Doctor of Medical Sciences' Dissertation*, Tbilisi, (in Russian)
17. Galperin E.I., Karagiulian S.R., Mochalov A.M. (1987) Experience of anatomical and atypical liver resections. *Khirurgiya*, No. 7, pp. 56–62 (in Russian)
18. Galperin E.I., Dederer Yu.M. (1987) *Nonstandard situations in hepatobiliary surgery*. Moscow: Metitsina Publishers, 336 pp. (in Russian)
19. Veronsky G.I. (1983) *Anatomophysiological aspects of liver resection*. Novosibirsk: Nauka Publishers, Siberian Branch, 185 pp. (in Russian)
20. Mochalov A.M. (1985) Ways of improving results of extensive liver resections. *Cand. of Med. Sci. Dissertation*, Moscow, 191 pp. (in Russian)
21. Zubov I.A. (1962) The pathomorphology and some problems of the pathogenesis of primary liver cancer in human opisthorchosis. *Author's abstract of Cand. of Med. Sci. Dissertation*, Tomsk, (in Russian)
22. Granov A.M. and Petrovichev N. N. (1977) *Primary liver cancer*. Leningrad: Meditsina Publishers, 224 pp. (in Russian)
23. Shain A.A. (1971) Opisthorchosis and cancer of the liver among the population of the Khanty-Mansy national district. *Vopr. onkologii*, No. 6, pp. 34–38 (in Russian)
24. Shain A.A. (1972) Specific clinical features of primary liver cancer *Klinicheskaya Meditsina*, No. 3, pp. 34–41 (in Russian)
25. Zhuravlev V.A. (1986) *Extensive and very extensive liver resections*. Saratov University Press, 214 pp. (in Russian)
26. Abasov B.Kh., Ashurov B.M., Gajiyev D.N. and Jumshudov D. (1987) Surgical treatment for the hydatid disease of the liver. In *Diagnostika i lecheniye ekhinokokkoza* (Diagnostics and treatment of the hydatid disease). Abstracts of National Scientific Conference, Baku, pp. 51–52 (in Russian)
27. Deineka I.Ya.(1968) *Echinococcosis in man*, Moscow: Meditsina Publishers, pp. 60–127 (in Russian)
28. Nikhinson R.A., Lubensky Yu.M. and Krakovsky A.I. (1990) Diagnostics and surgical treatment of focal lesions of the liver. *Khirurgiya pecheni*, Materials of a symposium attended by foreign specialists. February 13–14, Moscow, pp. 88–89 (in Russian)
29. Poluektov L.V., Rudakov V.A., Dobrovolsky A.I., Nekrasov D.S., Voronov O.E., Makushin D.G. *et al.* Surgical treatment of focal lesions of the liver. *Ibid.*, pp. 68–69 (in Russian)
30. Gilevich Yu.S., Rusakov V.I., Gabruashvili L.G., Gilevich M.Yu., Bodulin A.V. and Shcherbakov I.G. (1987) Socio-economic aspects of treating the hydatid disease. *Diagnostika i lecheniye ekhinokokkoza* (Diagnostics and treatment of the hydatid disease). Abstracts of National Scientific Conference. October 12–13, Baku, pp. 91–92 (in Russian)
31. Vafin A.Z., Shcherbakov I.G. Prevention of relapses of the hydatid disease. *Ibid.*, pp. 73–74 (in Russian)
32. Galperin E.I. Nasirov F.N. and Karagiulian S.R. On the question of treating residual cavities following surgery for the hydatid disease. *Ibid.*, pp. 88–89 (in Russian
33. Babur A.A., Milonov O.B., Dmitriyev B.I. and Zhuravok A.I. Plastic adhesive treatment of residual cavities in surgery for the hydatid disease. *Ibid.*, pp. 65–66 (in Russian)
34. Veronsky G.I. Experience in treating the hydatid disease of the liver. *Ibid.*, pp. 77–78 (in Russian)
35. Volokh Yu.A. Semi-open echinococcotomy of the liver as a method for preventing relapses. *Ibid.*, pp.82–83 (in Russian)
36. Vitebsky Ya.D., Chernov V.F. and Robak N>K. Peculiarities of the localization and complications of the hydatid disease. *Ibid.*, pp. 78–80 (in Russian)
37. Vilyavin M.Yu. (1986) Diagnosis and treatment of focal lesions of the liver by means of computer tomography. *Authors abstract of Cand. of Med. Sci. Dissertation*. Moscow, 23 pp. (in Russian)

38. Kuzin M.I., Todua F.I., and Vilyavin M.Yu. (1985) Computer tomography in the diagnosis and treatment of focal lesions of the liver. In *XX Plenum pravleniya vsesoyuznogo nauchnogo obshchestva khirurgov* (20th Plenary Session of the Board of the National Scientific Society of Surgeons). Abstracts of papers. Lvov, pp. 158–159 (in Russian)
39. Kuzin M.I., Todua F.I., Danilov M.V. *et al.* (1986) Computer tomography in the diagnosis and treatment of surgical diseases of the liver. *Vestnik khirurgii*, No. 1, pp. 36–40 (in Russian)
40. Todua F.I., Vilyavin M.Yu. (1985) Computer tomography in the diagnosis and treatment of surgical diseases of the liver. In *Vozmozhnosti i perspektivy kompyuternoi tomografii v diagnostike i lechenii khirurgicheskekh zabolevanij* (Opportunities and prospects of computer tomography in diagnosing and treating surgical diseases). Materials of an international symposium. Moscow, pp. 79–87 (in Russian)
41. Alperovich B.I., Yaroshkina T.N. (1990) US diagnostics of focal lesions of the liver and indications for surgery. In *Khirurgiya pecheni* (Hepatic Surgery). Materials of a symposium attended by foreign specialists. February 13–14, Moscow, pp. 3–4 (in Russian)
42. Shkrob O.S., Lotov A.N., Vetshev P.S. and Andrianov V.N. Ultrasonic diagnostics and treatment of focal lesions of the liver. *Ibid.*, pp. 5–7 (in Russian)
43. Volynsky Yu.D. and Guseinov E.K. Angiography in the diagnostics of focal lesions of the liver and concomitant disorders of portal circulation. *Ibid.* pp. 12–13 (in Russian)
44. Karagiulian S.R. and Nasirov F.N. Stage-by-stage diagnostics of tumoral lesions of the liver. *Ibid.*, pp. 13–15 (in Russian)
45. Grushin Yu.V., Apsatarov E.A., Kasimov D.N., Nukenova G.M. and Ibadilyin A.S. Present-day diagnostics of cavernous hemangiomas of the liver. *Ibid.*, p. 25 (in Russian)
46. Lyulinsky D.M., Todua F.I., and Vilyavin M.Yu. A comparative analysis of the ultrasonic and computer-tomographic investigations in diagnosing focal lesions of the liver. *Ibid.*, p. 8 (in Russian)
47. Tsybyrne K.A. and Kabak A.F. (1987) A comparative evaluation of various methods of diagnosing the hydatid disease. In *Diagnostika i lecheniye ekhinokokkoza* (Diagnosing and treating the hydatid disease). Abstracts of National Scientific Conference. Baku, pp. 23–25 (in Russian)
48. Leikina E.S., Ballad N.E., Zorikhina V.I. and Starkova T.V. Immunological methods in the diagnostics of the hydatid and alveolar disease. *Ibid.*, pp. 27–29 (in Russian)
49. Alperovich B.I., Tskhai V.D. and Reznikov A.T. (1986) Complications following liver resection. *Khirurgiya*, No. 7, pp. 106–110 (in Russian)
50. Zhuravlev V.A. (1982) A method for the surgical treatment of focal diseases of the liver. In *Fiziologiya i khirurgiya pecheni* (The Physiology and Surgery of the Liver). Tomsk, pp. 52–53 (in Russian)
51. Rustamov I.R. (1980) The rationale of surgical methods for the treatment of focal lesions of the liver. *Author's abstract of Doctor of Medicine Dissertation.* Kiev, 22 pp (in Russian)
52. Galperin E.I., Karagiulian S.R. and Nasirov F.N. (1990) Ways of reducing traumatism in liver resections. In *Khirurgiya pecheni* (Hepatic Surgery). Materials of a symposium attended by foreign specialists. February 13–14, Moscow, pp. 62–64 (in Russian)
53. Shapkin V.S. Peculiarities of resections and other operations in focal lesions of the liver. *Ibid.*, pp. 55–56 (in Russian)
54. Izraelashvili M.Sh. Topographoanatomical and surgical substantiation of liver resection for primary cancer. *Ibid.*, pp. 81–82 (in Russian)
55. Izraelashvili M.Sh., and Toidze Sh.S. Liver resection with the use of an inflatable bulb catheter. *Ibid.*, pp. 79–80 (in Russian)
56. Toidze Sh.S. and Izraelashvili M.Sh. (1984) *The use of transcatheter occlusion of the portal triad for controlling haemorrhage in liver resection.* Tbilisi, 18pp. (in Russian)
57. Galperin E.I. and Karagiulian S.R. (1989) A new simplified method of selective exposure of hepatic pedicles for controlled hepatectomies. *HPB Surgery*, **1**, pp. 119–130
58. Sorochenko O.A., Goloborodko N.K., Arnoldi E.K. and Lesovoi V.N. (1986) The bioactive method of electrosurgery of parenchymatous organs (experimental investigation). In *Khirurgich. lecheniye portalnoi gipertenzii, zabolev. i travm pecheni* (Surgical treatment of portal hypertention, diseases and traumas of the liver). Abstracts of papers. Kharkov, May 22–23, pp. 125–126 (in Russian)
59. Loshchilov V.I., Lykakhin A.A and Nevsky D.I. (1986) Ultrasonic aspirator for neurosurgery. In *Problemy tekhniki v meditsine* (Problems of technology in medicine). Abstracts of papers, IV National Scientific Conference, Tbilisi, Part I, pp. 115–116 (in Russian)
60. Alperovich B.I., Paramonova L.M. and Merzlikin N.V. (1985) *Cryosurgery of the liver and pancreas.* Tomsk, 124 pp. (in Russian)

61. Brekhov E.I., Skobelkin O.K., Tartynsky S.I., Rebizov V.Yu., Pekshev A.V. *et al.* (1988) The possibility of utilizing plasma flows for liver surgery. In *Abstracts of Papers at 21st plenary session of the Board of the National Scientific Society of Surgeons*. Krasnodar, pp. 96–97 (in Russian)
62. Koshelev V.N., Chalyk Yu.V. and Davydov N.Ya. $CO_2$ laser in hepatic surgery. *Ibid.*, pp. 112–113 (in Russian)
63. Skobelkin O.K., Litvin G.D., Ryabov V.I., Kirpichev A.G., Utkin V.V. *et al.* Specific features of hepatic surgery by means of laser irradiation. *Ibid.*, pp. 129–130 (in Russian)
64. Rustamov I.R., Shamuradov T.A. and Kurbaniyazov Z.B. (1990) Laser used for surgical treatment of hydatid disease of the liver. In *Khirurgiya pecheni* (Hepatic Surgery), Materials of a symposium attended by foreign specialists. February 13–14, Moscow, pp. 117–118 (in Russian)
65. Vakhidov A.V., Kalish Yu.I. and Ilkhamov F.A. The possibilities of laser irradiation and plasma scalpel in surgery for hydatid disease of the liver. *Ibid.*, pp. 118–119 (in Russian)
66. Babajanov B.R., Litvin G.D. and Eshchanov A.R. Laser and plasma scalpel in surgery for the hydatid disease of the liver. *Ibid.*, pp. 120–121 (in Russian)
67. Grubnik V.V., Melnik L.A., Grubnid Yu.V. and Fomichev A.A. The use of laser irradiation for hepatic surgery.*Ibid.*, pp. 137–138 (in Russian)
68. Korolev V.I. (1979) Harvesting the left liver lobe for heterotopic transplantation: topographoanatomical and experimental investigation. *Dissertation for Cand. of Med. Sci.*, Moscow, (in Russian)
69. Kuzin M.I., Todua F.I., Danilov M.V., Vishnevsky V.A. and Vilyavin M.Yu. (1986) Computer tomography in the diagnosis and treatment of surgical diseases of the liver. *Vestnik khirurgii im. I.I. Grekova*, No. 1, pp. 36–40 (in Russian)
70. Krylov N.L., Vyazitsky P.O., Seleznev Yu.K. *et al.* Targeted percutaneous punctures of pathological foci of the liver under control of computer tomography and ultrasonic examination. *Ibid.*, pp. 32–35 (in Russian)
71. Krylov N.L., Vyazitsky P.O. and Seleznev Yu.K. (1987) The use of computer tomography and echography in the diagnosis and treatment of abscesses of organs of the abdominal cavity and the retroperitoneal space. *Ibid.*, No. 9, pp. 41–46 (in Russian)
72. Shalimov S.A. YatsenkoL.D., Trokhimchenko E.P., Keisevich L.F., Volchenskova I.I. and Nikishin L.F. (1990) Radiosurgical methods of treating malignant tumours of the liver. In *Khirurgiya pecheni* (Hepatic Surgery). Materials of a symposium attended by foreign specialists. February 13–14, Moscow, pp. 37–38 (in Russian)
73. Guseinov E.K., Volynsky Yu.D. The role of radioendovascular surgery in the treatment of focal lesions of the liver. *Ibid.*, pp. 39--40 (in Russian)
74. Skuba N.D., Zelenov G.G. Vishnevsky V.A., and Guseinov E.K. Morphological changes in hemangiomas of the liver following endovascular occlusion of the branches of the hepatic artery during clinical management. Ibid., pp. 40–42 (in Russian)
75. Shalimov A.A., Nikishin L.F. and Keisevich L.V. Radioendovascular surgery for complicated cirrhosis of the liver. *Ibid.*, pp. 42–43 (in Russian)
76. Granov A.M., Noskov A.A. Ryzhkov V.K., Tarazov P.K., Derkach V.Yu. *et al.* Radioendovascular surgery as an independent or preparatory stage in the surgical treatment of cirrhosis of the liver, *Ibid.*, pp. 43–44 (in Russian)
77. Nazyrov F.G., Devyatov A.V., Murtayev N.M., Asabayev A.Sh. and Akilov Kh.A. Radioendovascular surgery for the treatment of aggravated cirrhosis of the liver. *Ibid.*, pp. 44–45 (in Russian)
78. Zemlyanoi A.G., Borisov A.E., Levin L.A., Zemlyanoi V.P., Borisova N.A. *et al.* Contemporary aspects of endovascular surgery for cirrhosis of the liver. *Ibid.*, pp. 49–50 (in Russian)
79. Ryzhkov V.K., Borisova N.A., Gapchenko E.M., and Dmitriyeva I.A. (1986) Spiral emboli for arterial occlusion. *Vestnik khirurgii im. I.I. Grekova*, No. 1, pp. 11–14 (in Russian)
80. Shumakov V.I., Galperin E.A., Zhuravlev V.A., Neklyudova E.A., Korolev V.I. *et al.* (1977) Transplantation of the left lobe of the liver: anatomical investigation. *Khirurgiya*, No. 3, pp. 43–49 (in Russian)
81. Karagiulian S.R. (1978) Methods of heterotopic transplantation of the left lobe of the liver: topographoanatomical and experimental investigation. *Dissertation for Cand. of Med. Sci.* Moscow, 161 pp. (in Russian)
82. Shumakov V.I., Galperin E.I., Neklyudova E.A., Mikhailov A.T., Korolev V.I. *et al.* (1978) Transplantation of the left lobe of the liver in experiment and in a clinical setting. *Khirurgiya*. No. 6, pp. 22–29 (in Russian)
83. The same authors. (1978) Transplantation of the left lobe of the liver in a clinical setting. In *Transplantatsiya organov v klinike i eksperimente i iskusstvennye organy* (Transplantation of Organs in a Clinical Setting and in Experiment and Artificial Organs). Moscow, pp. 11–16 (in Russian)

84. Dugladze D.I. (1984) Transplantation of the liver of the newborn to adult recipients: experimental investigation. *Dissertation for D.Sc.(Med.)*, Tbilisi, 307 pp. (in Russian)
85. Ioseliani G.D., Dugladze D.I., Girdaladze A.M. *et al.* (1982) Heterotopic transplantation of the liver of the newborn to an adult recipient. *Khirurgiya*, No. 1, pp. 52–56 (in Russian)
86. GalperinE.I. and Karagiulian S.R. (1984) Transplantation of the liver. In *Itogi nauki i tekhniki* (Results of Science and Technology) (VINITI) The Morphology of Man and Animals Series. Vol. 11, Transplantation of organs and tissues (Part 1). Moscow, pp. 5–70 (in Russian)

(*Accepted by S.Bengmark* 11 *September* 1990)

